# Effects of splenectomy on skin inflammation and psoriasis-like phenotype of imiquimod-treated mice

**DOI:** 10.1038/s41598-022-18900-7

**Published:** 2022-08-30

**Authors:** Hiroyo Shinno-Hashimoto, Akifumi Eguchi, Akemi Sakamoto, Xiayun Wan, Yaeko Hashimoto, Yuko Fujita, Chisato Mori, Masahiko Hatano, Hiroyuki Matsue, Kenji Hashimoto

**Affiliations:** 1 Department of Dermatology, Chiba University Graduate School of Medicine, Chiba, 260-8670 Japan; 2 Division of Clinical Neuroscience, Chiba University Center for Forensic Mental Health, Chiba, 260-8670 Japan; 3 Department of Sustainable Health Science, Center for Preventive Medical Sciences, Chiba University, Chiba, 263-8522 Japan; 4 Department of Biomedical Science, Chiba University Graduate School of Medicine, Chiba, 260-8670 Japan; 5 Department of Respirology, Chiba University Graduate School of Medicine, Chiba, 260-8670 Japan; 6 Department of Bioenvironmental Medicine, Graduate School of Medicine, Chiba University, Chiba, 260-8670 Japan

**Keywords:** Immunological disorders, Skin diseases

## Abstract

Imiquimod (IMQ) is widely used as animal model of psoriasis, a chronic inflammatory skin disorder. Although topical application of IMQ to back skin causes splenomegaly in mice, how the spleen affects the psoriasis-like phenotype of IMQ-treated mice remains unclear. In this study, we analyzed the cellular composition of spleen and measured metabolites in blood of IMQ-treated mice. We also investigated whether splenectomy influences the degree of skin inflammation and pathology in IMQ-treated mice. Flow cytometry showed that the numbers of CD11b^+^Ly6c^+^ neutrophils, Ter119^+^ proerythroblasts, B220^+^ B cells, F4/80^+^ macrophages, and CD11c^+^ dendritic cells in the spleen were significantly higher in IMQ-treated mice compared to control mice. An untargeted metabolomics analysis of blood identified 14 metabolites, including taurine and 2,6-dihydroxybenzoic acid, whose levels distinguished the two groups. The composition of cells in the spleen and blood metabolites positively correlated with the weight of the spleen. However, splenectomy did not affect IMQ-induced psoriasis-like phenotypes compared with sham-operated mice, although splenectomy increased the expression of interleukin-17A mRNA in the skin of IMQ-treated mice. These data suggest that the spleen does not play a direct role in the development of psoriasis-like phenotype on skin of IMQ-treated mice, though IMQ causes splenomegaly.

## Introduction

Psoriasis is an autoimmune disease that causes raised plaques and scaly patches on the skin. Yet psoriasis-induced inflammation could harm other organs: patients with psoriasis have a high risk for systemic comorbidities, including psoriatic arthritis, inflammatory bowel diseases, obesity, diabetes, and cardiovascular diseases. For example, approximately 30% of patients with psoriasis develop psoriatic arthritis^1^. Data from immunological and genetic studies suggest that interleukin-17 (IL-17) and IL-23 govern crosstalk between the innate and adaptive immune systems in a feed-forward amplification of psoriasis inflammation^2–4^. Although excessive activation of the immune system plays a crucial role in the pathogenesis of psoriasis, the precise mechanisms underlying this disease remain elusive^2–8^.

Imiquimod (IMQ), a Toll-like receptor 7 agonist, has been used as a rodent model of psoriasis^9,10^. It is also suggested that the skin serves as a peripheral neuroendocrine tissue^11,12^. Topical application of IMQ to back skin causes splenomegaly in rodents^9,13–17^. Given the key role of immune system in the spleen^18–21^, the spleen may play a role in psoriasis-like skin inflammation of IMQ-treated mice by modulating the immune system. However, how the spleen and splenectomy contribute to the psoriasis-like phenotype of IMQ-treated mice is unknown.

The present study investigated how the spleen impacts the psoriasis-like phenotype of IMQ-treated mice. First, we analyzed the cellular composition in the spleen of control and IMQ-treated mice by using flow cytometry. Then we examined correlations between spleen weight and cellular composition in the spleen. Second, we performed non-targeted metabolome analysis of blood samples from the two groups and examined correlations between spleen weight and blood metabolites. Finally, we determined whether splenectomy could affect psoriasis-like phenotype and skin inflammation in IMQ-treated mice.

## Results

### Effects of IMQ on weight and cell populations of spleen

**Figure 1:**
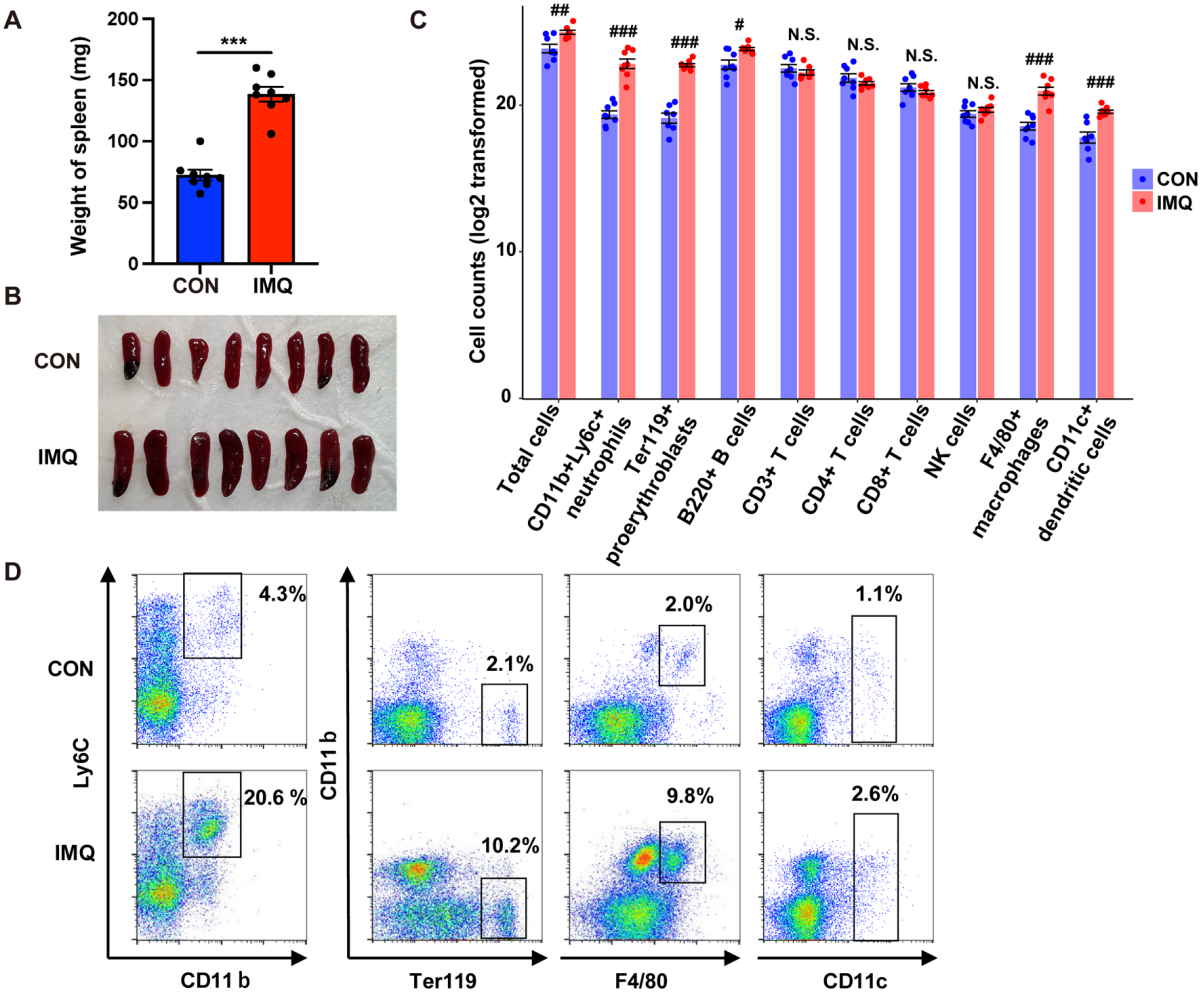
Effects of IMQ on weight and cell populations of spleen. (**A**): Spleen weight (Student t-test: df = 14, t = − 9.017, *P* = 0.000). (**B**): The photos of spleen from the control group and IMQ group. (**C**): FACS analysis of spleen samples from the two groups. Log2 transformed data of the number of total cells and each cell type. Total cells (Mann- Whitney test: U = 7, FDR-corrected *P* = 0.0079), CD11b^+^Ly6c^+^ neutrophils (Mann- Whitney test: U = 0, FDR-corrected *P* = 0.0005), Ter119^+^ proerythroblasts (Mann- Whitney test: U = 0, FDR-corrected *P* = 0.0006), B220^+^ B cells (Mann- Whitney test: U = 8, FDR-corrected *P* = 0.0105), CD3^+^ T cells (Mann- Whitney test: U = 19, FDR-corrected *P* = 0.2073), CD4^+^ T cells (Mann- Whitney test: U = 22, FDR-corrected *P* = 0.2073), CD8^+^ T cells (Mann- Whitney test: U = 19, FDR-corrected *P* = 0.1605), N.K1.1^+^ NK cells (Mann- Whitney test: U = 22.5, FDR-corrected *P* = 0.2073), F4/80^+^ macrophages (Mann- Whitney test: U = 0, FDR-corrected *P* = 0.0005), CD11c^+^ DCs (Mann- Whitney test: U = 1, FDR-corrected *P* = 0.0009). Data are shown as mean ± SEM (n = 8). One data of Ter119^+^ proerythroblasts and CD11c^+^ DCs in the control group and one data of CD3^+^ T cells in the IMQ group were missing because of technical problems. ^#^*P* (FDR-corrected) < 0.05, ^##^*P* (FDR-corrected) < 0.01, ^###^*P* (FDR-corrected) < 0.001. NS: not significant. (D): The representative FACS data of CD11b^+^Ly6c^+^ neutrophils, Ter119^+ ^proerythroblasts, F4/80^+^ macrophages, and CD11c^+^ DCs in the spleen of control mice and IMQ-treated mice.

Topical application of IMQ caused significantly increased spleen weight in IMQ-treated mice compared to control mice (Fig. 1A,B), consistent with our previous report^22^. The number of total cells of IMQ-treated mice was also significantly higher than those of control mice (Fig. 1C). Spleen cells were analyzed for the percentage and the number of CD11b^+^Ly6c^+^ neutrophils, Ter119^+^ proerythroblasts, B220^+^ B cells, CD3^+ ^T cells, CD4^+^ T cells, CD8^+^ T cells, NK1.1^+^ natural killer (NK) cells, F4/80^+^ macrophages, and CD11c^+^ dendritic cells (DCs). The number of neutrophils, proerythroblasts, B cells, macrophages, and DCs in the spleen of IMQ-treated mice was significantly higher than those of control mice (Fig. 1C,D). In contrast, there were no differences in the number of T cells (CD3^+^, CD4^+^, and CD8^+^) and NK cells (Fig. 1C).

**Figure 2:**
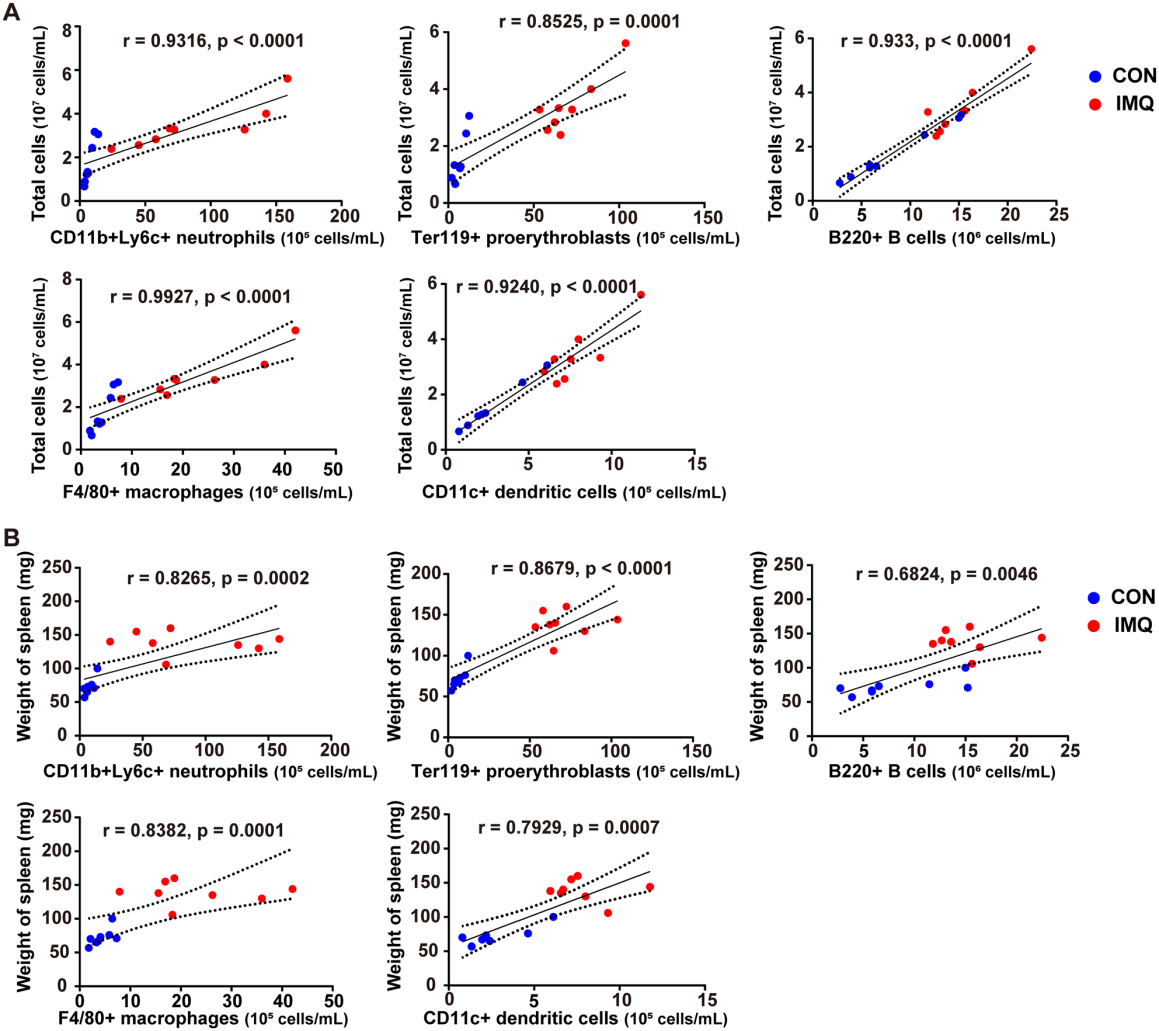
Correlations between the number of total splenic cells (or spleen weight) and the number of the splenic cell types. (**A**): The correlations between the number of total splenic cells and the number of the splenic cell types whose amount significantly increased in IMQ group. (**B**): The correlations between the weight of spleen and the number of the splenic cell types whose amount significantly increased in IMQ group. The values represent the mean ± SEM (n = 8).

The total number of cells in the spleen positively correlated with the cell types (i.e., neutrophils, proerythroblasts, B cells, macrophages, and DCs) in the two groups (Fig. 2A). Moreover, the weight of spleen positively correlated with the cell types (i.e., neutrophils, proerythroblasts, B cells, macrophages, and DCs) in the two groups (Fig. 2B).

### Non-targeted metabolomic profiling of plasma

**Figure 3:**
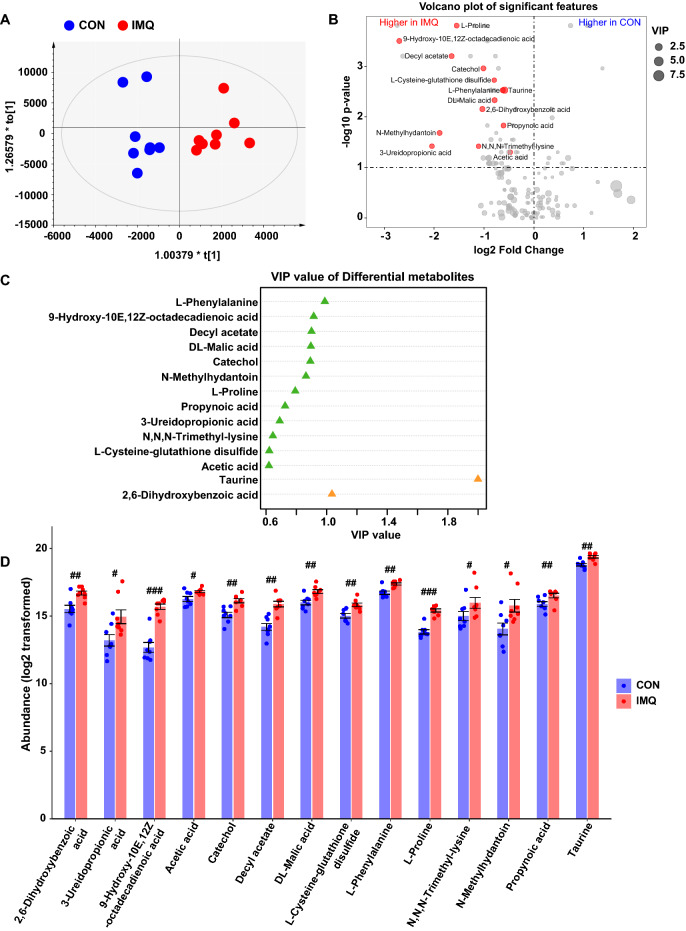
Effects of IMQ on metabolites of plasma. (**A**): Scatter plot of murine plasma metabolites based on orthogonal partial least square discriminant analysis (OPLS-DA) between IMQ group and control group. (**B**): Volcano plot shows the differential metabolites between the two groups. The X-axis indicates the log2-transformed plasma metabolite abundance of fold change, and the Y-axis indicates the -log10-transformed *P* value using the Wilcoxon rank sum test. Horizontal lines indicate *P* < 0.05. Increased or decreased metabolites are marked in red and blue, respectively. The size of the dot represents the size of the VIP value. Metabolites with *P* < 0.05 and VIP > 0.6 are mentioned in text. (**C**): VIP value of the differential metabolites between the two groups. (**D**): Log2 transformed data of the abundance of the differential plasma metabolites. 2,6-dihydroxybenzoic acid (Mann- Whitney test: U = 7, FDR-corrected *P* = 0.0047), 3-ureidopropionic acid (Mann- Whitney test: U = 12, FDR-corrected *P* = 0.0177), 9-hydroxy-10E,12Z-octadecadienoic acid (Mann- Whitney test: U = 1, FDR-corrected *P* = 0.0009), acetic acid (Mann- Whitney test: U = 13, FDR-corrected *P* = 0.0216), catechol (Mann- Whitney test: U = 3, FDR-corrected *P* = 0.0016), decyl acetate (Mann- Whitney test: U = 2, FDR-corrected *P* = 0.0013), DL-malic acid (Mann- Whitney test: U = 6, FDR-corrected *P* = 0.0035), L-cysteine-glutathione disulfide (Mann- Whitney test: U = 4, FDR-corrected *P* = 0.0023), L-phenylalanine (Mann- Whitney test: U = 5, FDR-corrected *P* = 0.0026), L-proline (Mann- Whitney test: U = 0, FDR-corrected *P* = 0.0009), *N,N,N*-trimethyl-lysine (Mann- Whitney test: U = 12, FDR-corrected *P* = 0.0177), *N*-methylhydantoin (Mann- Whitney test: U = 10, FDR-corrected *P* = 0.0114), propynoic acid (Mann- Whitney test: U = 9, FDR-corrected *P* = 0.0089), taurine (Mann- Whitney test: U = 5, FDR-corrected *P* = 0.0026). Data are shown as mean ± SEM (n = 8). ^#^*P* (FDR-corrected) < 0.05, ^##^*P* (FDR-corrected) < 0.01, ^###^*P* (FDR-corrected) < 0.001.

We performed non-targeted metabolomic profiling of plasma samples from IMQ-treated mice and control mice. After quality control and removal of low-abundance peaks, a subset of 173 metabolites was annotated. Orthogonal partial least squares discriminant analysis (OPLS-DA) revealed that the metabolic composition of IMQ group was significantly different from that of the control group (Fig. 3A). After thresholding (variable importance in the projection [VIP] value > 0.6, Wilcoxon rank p-value < 0.05), we identified 14 metabolites altered between the two groups (Fig. 3B). Among the 14 metabolites, taurine and 2,6-dihydroxybenzoic acid had VIP > 1.0 (Fig. 3C). The fourteen metabolites that increased in the IMQ group were taurine, 2,6-dihydroxybenzoic acid, L-phenylalanine, 9-hydroxy-10E,12Z-octadecadienoic acid, decyl acetate, DL-malic acid, catechol, *N*-methylhydantoin, L-proline, propynoic acid, 3-ureidopropionic acid, *N,N,N*-trimethyl-lysine, L-cysteine-glutathione disulfide, and acetic acid (Fig. 3D).

### Associations between spleen cells, spleen weight, and plasma metabolites

**Figure 4:**
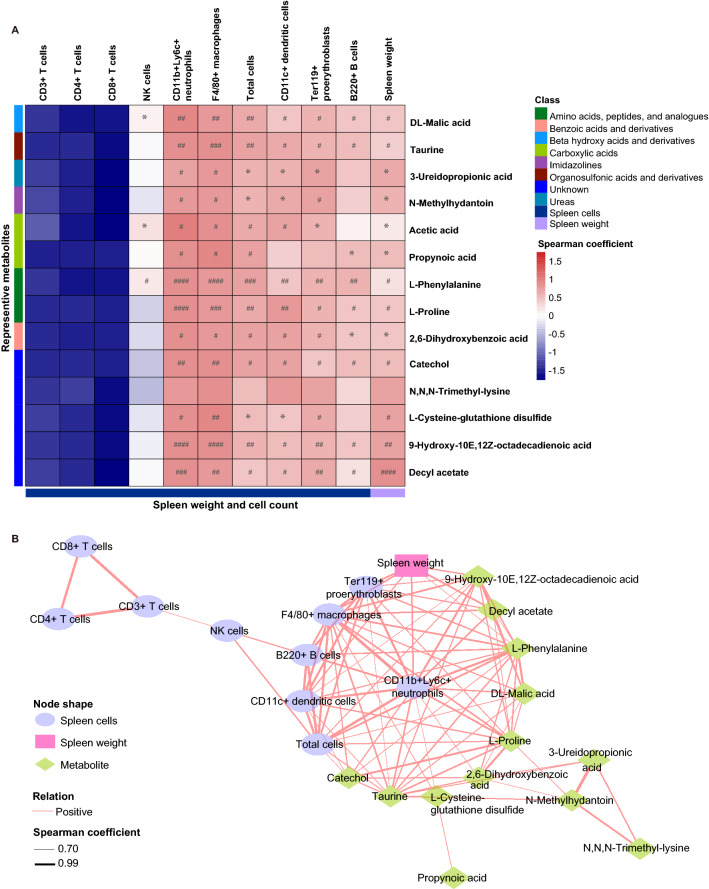
Associations among spleen cell types, spleen weight and the metabolites of plasma samples. (**A**): Heatmap of Spearman rank correlation coefficients between counts of splenic cell types or spleen weight and the metabolites of plasma samples. *P < 0.05 and FDR-corrected *P* > 0.05, ^#^*P* (FDR-corrected) < 0.05, ^##^*P* (FDR-corrected) < 0.01, ^###^*P* (FDR-corrected) < 0.001, ^####^*P* (FDR-corrected) < 0.0001. (**B**): Correlation network analysis reveals the associations among counts of spleen cell types, spleen weight and the plasma metabolites. Each node shape represents spleen cell types, spleen weight or plasma metabolites, respectively. The pink lines connecting the nodes indicate positive correlation and the line weight indicates spearman correlation coefficient. We created the network using Cytoscape software 3.8.0. (https:cytoscape.org).

Spearman correlation analysis was used to quantify the correlations between the spleen cell types, spleen weight, and the fourteen differential metabolites of plasma. Several cell types and spleen weight were significantly correlated with the plasma metabolites in the two groups (Fig. 4A). The cell types such as total cells, neutrophils, and macrophages were positively associated with plasma metabolites except *N,N,N*-trimethyl-lysine. The other cell types (B cells, proerythroblasts, and DCs) were positively associated with several metabolites. Spleen weight was also positively associated with plasma metabolites except *N,N,N*-trimethyl-lysine (Fig. 4A).

Spearman correlation was also used to determine if IMQ-related cell types in the spleen and plasma metabolites contribute to splenomegaly. The cell types in the spleen and plasma metabolites differentially abundant in the two groups showed more associations with spleen weight (Fig. 4B). Interestingly, neutrophils in the spleen were positively correlated with spleen weight and plasma metabolites (Fig. 4B).

### Effect of splenectomy on skin inflammation and body weight changes in IMQ-treated mice

**Figure 5:**
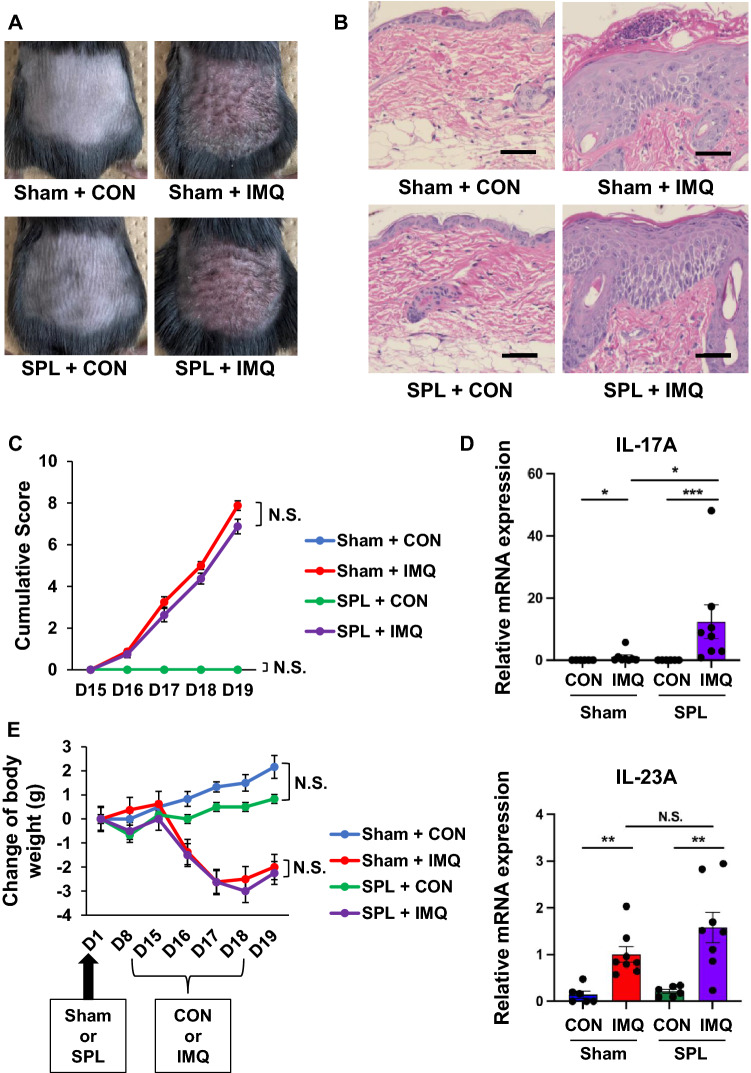
Effect of splenectomy on the skin inflammation and body weight changes in IMQ-treated mice. (**A**): Mice were treated topically with 5% IMQ cream or control cream on the shaved back for four days two weeks after sham or splenectomy. The representative photos of back skin from the four groups. (**B**): HE staining of the back skin. Scale bar = 50 μm. (**C**): Cumulative skin scores from day 15 to day 19. Day 15 (Kruskal–Wallis test: H = 0.000, *P* = 1.000), day 16 (Kruskal–Wallis test: H = 17.792, *P* = 0.000), day 17 (Kruskal–Wallis test: H = 22.840, *P* = 0.000), day 18 (Kruskal–Wallis test: H = 23.619, *P* = 0.000), day 19 (Kruskal–Wallis test: H = 23.459, *P* = 0.000). Scores of sham + control group and splenectomy + control group are 0 from D15 to D19. (**D**): Relative mRNA expression levels of IL-17A and IL23-A in murine back skin. IL-17A mRNA (Kruskal–Wallis test: H = 23.332, *P* = 0.000), IL-23A mRNA (Kruskal–Wallis test: H = 19.302, *P* = 0.000). *P < 0.05, **P < 0.01, ***P < 0.001. (**E**): Change of body weight. Day 1 (Kruskal–Wallis test: H = 0.000, *P* = 1.000), day 8 (Kruskal–Wallis test: H = 7.641, *P* = 0.054), day 15 (Kruskal–Wallis test: H = 2.022, *P* = 0.568), day 16 (Kruskal–Wallis test: H = 10.921, *P* = 0.012), day 17 (Kruskal–Wallis test: H = 20.213, *P* = 0.000), day 18 (Kruskal–Wallis test: H = 21.758, *P* = 0.000), day 19 (Kruskal–Wallis test: H = 20.287, *P* = 0.000). The values represent the mean ± SEM. (n = 6 or 8). NS: not significant. SPL: Splenectomy.

Topical application of IMQ induced psoriasis-like dermatitis in both sham group and splenectomy group (Fig. 5A). Representative hematoxylin and eosin staining of the back skin showed acanthosis and parakeratosis with microabscess in the two groups (Fig. 5B). The cumulative scores of the sham-IMQ group and splenectomy-IMQ group were not significantly different (Fig. 5C).

Next, we measured the gene expression levels of IL-17A and IL-23A in the skin. The mRNA levels of IL-17A and IL-23A in the IMQ group were higher than those of the control group (Fig. 5D). Expression of IL-17A mRNA in the splenectomy-IMQ group was significantly higher than that of sham-IMQ group, whereas the expression of IL-23A mRNA was not different between the sham-IMQ group and splenectomy-IMQ group (Fig. 5D). There were also no changes in the body weight between sham group and splenectomy group in both of IMQ group and control group (Fig. 5E).

## Discussion

This is the first study to show how the spleen contributes to psoriasis-like phenotypes in IMQ-treated mice. Indeed, topical application of IMQ significantly increased infiltration of immune cells such as neutrophils, DCs, macrophages, and B cells in the spleen, which agrees with previous studies. These cell types correlated with spleen weight, which also correlated with higher levels of 14 metabolites in the plasma of IMQ-treated mice; taurine and 2,6-dihydroxybenzoic acid had especially high VIP values. These metabolites likely contribute to splenomegaly upon IMQ application. Finally, we showed that splenectomy does not affect a psoriasis-like phenotype on the skin of IMQ-treated mice but could potentiate IMQ-induced increases in IL-17A mRNA in the skin.

Neutrophils, proerythroblasts, B cells, macrophages, and DCs significantly enlarged the IMQ-treated spleen; their cell compartments positively correlated with spleen weight. Interestingly, the percentage of B cells in the spleen did not change between the control group and IMQ-treated group. Previously, percentages of macrophages and DCs increased and the percentage of T cells (both CD4^+^ and CD8^+^) decreased in an IMQ-treated group^9^, consistent with the current data. Topical treatment with IMQ likely induces inflammation, and increased numbers of immune cells cause splenomegaly.

The longitudinal diameter of the spleen and the duration of psoriasis were correlated in a previous study of 79 psoriatic patients^23^, suggesting that increased diameter of the spleen in psoriatic patients with long-term illness is related to chronic inflammation. Future clinical studies are needed to confirm the role of spleen in the pathogenesis of psoriasis^24^.

Non-targeted metabolomics profiling identified 14 metabolites whose levels were significantly different between the IMQ-treated group and the control group. Taurine and 2,6-dihydroxybenzoic acid had the highest VIP values, indicating that they contributed the most to the separation between groups. Taurine plays an important role in inflammation associated with oxidative stress^25,26^ and may play a pathological role in psoriasis given its higher levels in blood from patients with psoriasis when compared to healthy controls^27^. Taurine reduced blood levels of IL-6 in patients with traumatic brain injury in a recent randomized double-blind controlled trial^28^. Taurine's anti-inflammatory actions^25,26^ in the blood of IMQ-treated mice may compensate for IMQ-induced inflammation in the body. Further studies should explore how taurine affects spleen size in IMQ-treated mice. The function of 2,6-dihydroxybenzoic acid remains unclear but is likely anti-inflammatory^29^. Higher levels of 2,6-dihydroxybenzoic acid in the blood of IMQ-treated mice may also compensate for IMQ-induced inflammation in the body. These metabolites should be targeted in future studies on splenomegaly of IMQ-treated mice.

Spleen enlargement is linked to systemic inflammation^30^. For example, we reported splenomegaly in mice treated with lipopolysaccharide (LPS), and there were positive correlations between spleen weight and blood levels of pro-inflammatory cytokines (i.e., IL-6, tumor necrosis factor-α)^31–33^. A chronic social defeat stress (CSDS) model revealed that the spleen weight of susceptible mice with depression-like behaviors was higher than that of control mice and CSDS-resilient mice^34^. Collectively, it is likely that LPS- (or CSDS)-induced splenomegaly is associated with systemic inflammation^31–36^.

Considering the spleen's key role in the immune system^18–21^, we investigated how splenectomy affects psoriasis-like pathology and skin inflammation of IMQ-treated mice. Unexpectedly, splenectomy did not change the psoriasis-like phenotype in IMQ-treated mice. However, we found that splenectomy significantly enhanced IL-17A mRNA in the skin of IMQ-treated mice compared to sham-operated mice. The spleen may not directly impact the psoriasis-like phenotype of IMQ-treated mice, but it does cause splenomegaly.

This study has one limitation. In this study, we used 5% IMQ cream (Beselna cream). The full list of excipients is isostearic acid, benzyl alcohol, cetyl alcohol, stearyl alcohol, white soft paraffin, polysorbate 60, sorbitan stearate, glycerol, methyl hydroxybenzoate, propyl hydroxybenzoate, xanthan gum, and purified water. Walter et al.^37^ reported that isostearic acid, a major component, could promote inflammasome activation in cultured keratinocytes, and that it increased the expression of inflammatory cytokines in vivo. These data suggest that isostearic acid may contribute to the observed effects of Beselna cream used in this study^37,38^. In this study, we did not examine the effects of isosteatic acid on spleen function since the company did not disclose the detailed information of excipients including isostearic acid. Further study is needed to investigate the effects of isostearic acid on spleen functions.

In conclusion, this study highlighted the key role of the spleen in chronic inflammation of IMQ-treated mice. The numbers of neutrophils, proerythroblasts, B cells, macrophages, and DCs in the spleen significantly increased, which correlated with higher spleen weight. Metabolomics profiling also revealed metabolites whose roles in psoriasis pathogenesis can be studied further. However, splenectomy did not affect psoriasis-like phenotypes in IMQ-treated mice. Although the spleen may not play a major role in psoriasis-like phenotypes in IMQ-treated mice, topical application of IMQ to back skin causes splenomegaly.

## Materials and methods

### Animals

Nine-week-old female C57BL/6 mice (weighing 18–21 g, n = 16, Japan SLC Inc., Hamamatsu, Shizuoka, Japan) were used in Experiment 1. Seven-week-old female C57BL/6 mice (weighing 18–21 g, n = 28, Japan SLC Inc., Hamamatsu, Shizuoka, Japan) were used in Experiment 2. Mice were housed (3–4 per cage) under a 12-h/12-h light/dark cycle (lights on between 07:00 and 19:00), with ad libitum access to food (CE-2; CLEA Japan, Inc., Tokyo, Japan) and water. The experimental protocol was approved by the Chiba University Institutional Animal Care and Use Committee (Permission number: 2–433). All procedures were performed in accordance with the relevant guidelines and regulations, and the study complied with ARRIVE (Animal Research: Reporting of In Vivo Experiments) guidelines. All efforts were made to minimize animal suffering^22^.

### IMQ treatment

The shaved back skin of mice was treated with 62.5 mg of 5% IMQ cream (Beselna cream; Mochida Pharmaceutical Co., Tokyo, Japan) daily for four consecutive days as previously described^22^. Control mice were treated similarly with 62.5 mg of white petrolatum (Maruishi Pharmaceutical Co., Osaka, Japan).

### Splenectomy

Splenectomy (or sham) surgery was performed under continuous isoflurane inhalation anesthesia as previously described^39^. Briefly, the mice were anesthetized with 3% isoflurane through an inhalation anesthesia apparatus (KN-1071NARCOBIT-E; Natsume Seisakusho, Tokyo, Japan). In the splenectomy group, each mouse was maintained in a right lateral recumbent position, and an approximately 1-cm incision was made from the abdominal wall under the left costal margin. The skin was dissected, and subcutaneous, muscle, and fascia layers were removed individually until the spleen was exposed. The peripheral ligament of the spleen was separated, associated blood vessels and nerves were ligatured using 6–0 silk sutures, and the spleen was removed by transecting the blood vessels distal to the ligature. Abdominal muscles and the skin incision were closed sequentially using 4–0 silk sutures. The abdominal wall was similarly opened during sham surgery, and the wall was closed immediately after identifying the spleen^39^. In this study, we did not use opioid and/or non-steroidal anti-inflammatory drugs for pain management after surgery.

### Sample collection

#### Experiment 1

 After IMQ treatment for four consecutive days, the skin, spleen, and blood samples were collected on day 5.

#### Experiment 2

 Splenectomy or sham was carried out on day 1. Mice were treated with IMQ from day 15 to day 18, and skin samples were collected on day 19. The clinical skin score was measured from day 15 to day 19. The degree of skin inflammation was assessed with a cumulative disease severity score, similar to the human Psoriasis Area and Severity Index but without considering the area. Erythema, scaling, and thickening were scored independently from 0 to 4: 0, none; 1, slight; 2, moderate; 3, marked; 4, very marked. The single scores were summed; the highest possible score is 12^9^.

### Fluorescence activated cell sorting analysis of spleen samples

Mouse spleen tissues were mashed and passed through a 100-μm mesh to prepare a single cell suspension and treated with lysis buffer (0.155 M ammonium chloride, 0.1 M disodium EDTA, 0.01 M potassium bicarbonate) to lyse erythrocytes. Spleen cells were suspended and counted using an automated cell counter (BIO-RAD, Alfred Nobel Drive, CA) prior to fluorescence activated cell sorting (FACS) analysis. We stained 10^6^ cells with various monoclonal antibodies against cell surface antigens for 30 min at 4 °C and then washed them with an FACS buffer [3% fetal calf serum (FCS), 0.04% NaN3 in phosphate-buffered saline]. Cells were resuspended with 0.4 μg/ml propidium iodine (cat# P-170: Sigma) containing FACS buffer. The following antibodies were used: anti CD11b-PE (× 400 diluted using FACS buffer, cat# 553,312: BD Bioscience, Franklin Lakes, NJ), anti Ly6c-FITC (× 100, cat# 553,104: BD Bioscience), anti B220-PE (× 200, cat# 553,309: BD Bioscience), anti CD8alpha-allophycocyanin (× 100, cat# 553,035: BD Bioscience), anti NK1.1-PE (× 100, cat# 553,165: BD Bioscience), anti CD11c-PE (× 100, cat# 557,401: BD Bioscience), anti Ter119-PE (× 40, cat# 12–5921-83: eBioscience, San Diego, CA), anti CD4-allophycocyanin (× 100, cat# 17–0042-82: eBioscience, San Diego, CA), anti F4/80-PE (× 40, cat# 12–4801-80: Invitrogen), and anti CD3-FITC (× 40, cat# 100,305: BioLegend, San Diego, CA). The stained cells were analyzed using FACSCantII and FlowJo software (BD Bioscience).

### Untargeted metabolomics analysis of plasma samples

Untargeted metabolomics analysis was performed using an ExionLC AD UPLC system (SCIEX, Tokyo, Japan) interfaced with an X500R LC-QToFMS system (SCIEX, Tokyo, Japan) with electrospray ionization (ESI) operating in positive and negative ionization mode, as previous reported^40,41^. First, 100 μL of methanol containing internal standards (100 μM N*,N*-diethyl-2-phenylacetamide and d-camphor-10-sulfonic acid) was added to the plasma samples (100 μL), and then samples were centrifuged at 14,000 × rpm for 5 min. After centrifugation, the supernatant was transferred to an Amicon® Ultra-0.5 3 kDa filter column (Merck Millipore, Tokyo, Japan) and centrifuged at 14,000 × rpm for 1 h. The filtrate was transferred to glass vials for subsequent analysis.

The metabolomics data was analyzed with Mass Spectrometry-Data Independent AnaLysis (MS-DIAL) software version 4.60^42^ and R statistical environment Ver 4.0.5. Only metabolites present in 50% of the samples were measured, and metabolites whose coefficient of variation value was over 30% in pooled QC samples were removed from analysis. Annotation level 2 proposed by Schymanski et al.^43^ was used for data analysis.

### Histology

Back skin samples from control and IMQ-treated groups were collected and fixed in 10% formalin (FUJIFILM Wako Pure Chemical Corp., Tokyo, Japan). Staining with hematoxylin and eosin (HE) was performed at the Biopathology Institute Co., Ltd (Kunisaki, Oita, Japan) as previously reported^22^. Back skin samples were embedded in paraffin, and 3-μm sections were prepared and stained with HE. Representative images of two groups were obtained using a Keyence BZ-9000 Generation II microscope (Osaka, Japan) as previously reported^22^.

### Quantitative real-time polymerase chain reaction

RNA was isolated using TRIzol LS Reagent (Invitrogen, Carlsbad, CA, USA) according to the manufacturer’s instructions; cDNA was generated using the High-Capacity cDNA Reverse Transcription Kit (Applied Biosystems, Waltham, MA, USA) and TaKaRa polymerase chain reaction (PCR) Thermal Cycler Dice (Takara Bio Inc., Kusatsu, Shiga, Japan), and quantitative real-time PCR was performed using the StepOnePlus Real-Time PCR System (Applied Biosystems, Waltham, MA, USA). The mouse primers for glyceraldehyde-3-phosphate dehydrogenase (GAPDH) (4352339E), IL-17A (Mm00439618), and IL-23A (Mm00518984) were obtained from Applied Biosystems. The GAPDH housekeeping gene was used to normalize gene expression.

### Statistical analysis

Data are shown as the mean ± standard error of the mean. Data were analyzed using GraphPad Prism (Tokyo, Japan). Student's t-test was performed to compare spleen weights between the two groups. Spleen cell types and plasma metabolites were compared between the two groups using Mann–Whitney U-test with a false discovery rate (FDR) control. Correlations among spleen weight, spleen cells, and plasma metabolites were evaluated using Spearman’s correlation analysis. For multivariate analysis of the metabolome data, orthogonal partial least squares discriminant analysis (OPLS-DA) was performed in Simca-P V.14.0 (Umetrics AB). Metabolites with VIP > 0.6 and *p*-value < 0.05 (Wilcoxon rank-sum test) were considered differentially abundant. Cumulative skin score, relative mRNA expression of skin, and body weight changes were analyzed with a Kruskal–Wallis test. *P* < 0.05 (or FDR-corrected *P* < 0.05) was considered statistically significant.

## Data Availability

The data that support the findings of this study are available from the corresponding author upon reasonable request.
